# Systematic metabolic engineering of *Zymomonas mobili*s for β-farnesene production

**DOI:** 10.3389/fbioe.2024.1392556

**Published:** 2024-05-17

**Authors:** Yubei Xiao, Xuemei Tan, Qiaoning He, Shihui Yang

**Affiliations:** State Key Laboratory of Biocatalysis and Enzyme Engineering, School of Life Sciences, Hubei University, Wuhan, China

**Keywords:** β-farnesene synthase, *Zymomonas mobilis*, CRISPR–Cas system, 2-C-methyl-erythritol 4-phosphate pathway, carbon/nitrogen ratio, β-farnesene

## Abstract

*Zymomonas mobilis* is an ethanologenic bacterium that can produce hopanoids using farnesyl pyrophosphate (FPP), which can be used as the precursor by β-farnesene synthase for β-farnesene production. To explore the possibility and bottlenecks of developing *Z. mobilis* for β-farnesene production, five heterologous β-farnesene synthases were selected and screened, and *AaBFS* from *Artemisia annua* had the highest β-farnesene titer. Recombinant strains with *AaBFS* driven by the strong constitutive promoter P*gap* (P*gap*–*AaBFS*) doubled its β-farnesene production to 25.73 ± 0.31 mg/L compared to the recombinant strain with *AaBFS* driven by P*tet* (P*tet*–*AaBFS*), which can be further improved by overexpressing the P*gap*–*AaBFS* construct using the strategies of multiple plasmids (41.00 ± 0.40 mg/L) or genomic multi-locus integration (48.33 ± 3.40 mg/L). The effect of cofactor NADPH balancing on β-farnesene production was also investigated, which can be improved only in *zwf*-overexpressing strains but not in *ppnK*-overexpressing strains, indicating that cofactor balancing is important and sophisticated. Furthermore, the β-farnesene titer was improved to 73.30 ± 0.71 mg/L by overexpressing *dxs*, *ispG*, and *ispH*. Finally, the β-farnesene production was increased to 159.70 ± 7.21 mg/L by fermentation optimization, including the C/N ratio, flask working volume, and medium/dodecane ratio, which was nearly 13-fold improved from the parental strain. This work thus not only generated a recombinant β-farnesene production *Z. mobilis* strain but also unraveled the bottlenecks to engineer *Z. mobilis* for farnesene production, which will help guide the future rational design and construction of cell factories for terpenoid production in non-model industrial microorganisms.

## Graphical Abstract

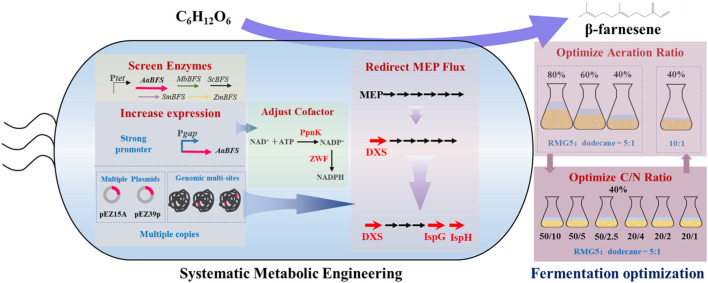

## Highlights

• Five β-farnesene synthases screened with *AaBFS* were selected for β-farnesene production in *Zymomonas mobilis.*
• β-Farnesene production was improved by increasing the expression of *AaBFS* using a strong promoter or multiple copies through multiple plasmid constructs or genomic multi-locus integration.
• 2-C-Methyl-erythritol 4-phosphate (MEP) flux enhancement was crucial for increasing β-farnesene production by enhancing the expression of key enzymes of DXS, IspG, and IspH.
• β-Farnesene production was improved by optimizing aeration and C/N ratios.
• The highest β-farnesene titer of 159.70 mg/L was achieved for recombinant *Z. mobilis* in flask fermentation.

## 1 Introduction

As an important sesquiterpene organic compound, farnesene has two major isomers of α- and β-farnesene in nature. In particular, β-farnesene has been identified as an aphid alarm pheromone, which was first applied for pest control in agricultural protective agents (Tang et al., 2021; Liu et al., 2022a). β-Farnesene is also an ideal substitute for sustainable aviation fuels (SAFs) with characteristics of high efficiency, high calorific value, and being green renewable. The appropriate cetane numbers, density, and low cloud points also enable hydrogenated β-farnesene to be used for diesel fuel (Liu et al., 2022a). Moreover, β-farnesene exhibits excellent characteristics as a raw material in industrial production, such as lubricants, surfactants, and cosmetics. Additionally, β-farnesene has also been employed as an intermediate for vitamin E biosynthesis, which can reduce carbon emissions by 60%, showing advantages in environmental protection compared to traditional chemical synthesis (Chen et al., 2018; Ye et al., 2022).

Farnesene can be produced through chemical synthesis, but the inevitable problems of environmental pollution and high production cost due to the impurity of different isomers and byproducts hinder its development (Bi et al., 2022a). Alternatively, the biological route requires extensive efforts to engineer microorganisms such as *Escherichia coli* and *Saccharomyces cerevisiae* for β-farnesene production using systematic metabolic engineering approaches (Meadows et al., 2016; Wang et al., 2021; Bi et al., 2023).

**FIGURE 1:**
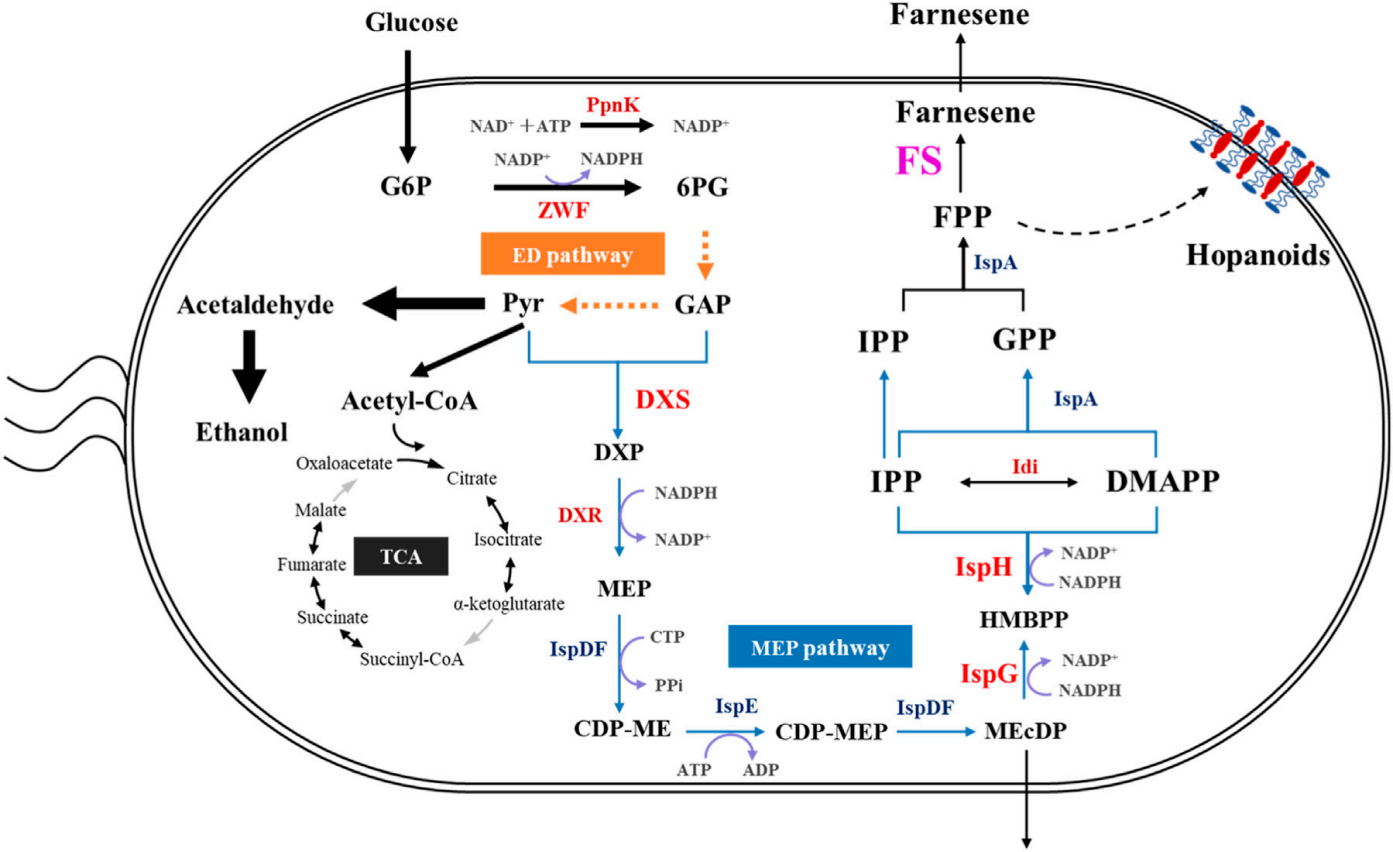
Schematic diagram of the metabolic pathway and the key genes for constructing the efficient farnesene production recombinant strain of *Z. mobilis*. CDP-ME, 4-diphosphocytidyl-2-C-methyl-D-erythritol; CDP-MEP, 4-diphosphocytidyl-2-C-methyl-D-erythritol 2-phosphate; DMAPP, dimethylallyl diphosphate; DXS, 1-deoxy-D-xylulose 5-phosphate synthase; DXP, 1-deoxy-D-xylulose 5-phosphate; DXR, 1-deoxy-D-xylulose 5-phosphate reductoisomerase; ED, Entner–Doudoroff pathway; FPP, farnesyl pyrophosphate; GPP, geranyl pyrophosphate; GAP, glyceraldehyde 3-phosphate; G6P, glucose 6-phosphate; 6 PG, gluconolactone 6-phosphate; HMBPP, 4-hydroxy-3-methylbut-2-enyl-diphosphate; Idi, IPP and DMAPP isomerase; IPP, isopentenyl diphosphate; IspD, 2-C-methyl-D-erythritol 4-phosphate cytidylytransferase; IspE, 4-diphosphocytidyl-2-C-methyl-D-erythritol kinase; IspDF, bifunctional enzyme with CDP-ME synthase and MEcDP synthase; IspF, 2-C-methyl-D-erythritol 2,4-cyclodiphosphate synthase; IspG, HMBPP synthase; IspH, HMBPP reductase; MEcDP, 2-C-methyl-D-erythritol 2,4 cyclodiphosphate; MEP, 2-C-methyl-D-erythritol 4-phosphate; PpnK, NAD^+^ kinase; Pyr, pyruvate; ZWF, glucose-6-phosphate dehydrogenase.

There are two natural and distinct metabolic pathways, namely, the 2-C-methyl-erythritol 4-phosphate (MEP) and mevalonate (MVA) pathways, for the biosynthesis of isopentenyl pyrophosphate (IPP) and dimethylallyl pyrophosphate (DMAPP), which are the precursors of farnesene. Usually, the MEP pathway is present in eubacteria, algae, cyanobacteria, and apicomplexan parasites, whereas the MVA pathway mainly exists in the cytosol and mitochondria of plants and fungi, archaea, and eukaryotes (Liu et al., 2022a). IPP and DMAPP can be condensed together to obtain farnesyl pyrophosphate (FPP), which is the precursor for β-farnesene biosynthesis through farnesene synthase (FS) (Figure 1).

Considerable systematic metabolic engineering efforts have been devoted to increase the farnesene yield in various microbial chassis cells (Tang et al., 2021; Liu et al., 2022a), including promoting the precursor supply (Rico et al., 2019), improving the cofactor supply (Liu et al., 2014), enhancing the biosynthesis of IPP and DMAPP by overexpressing the key enzymes of MEP (Appanna et al., 2012; Liu et al., 2014) or MVA pathways (Meadows et al., 2016; Ye et al., 2022), and enabling MVA pathway compartmentalization (Yao et al., 2020) to optimize the FPP flux (Lim et al., 2020). In addition, the balance between IPP and DMAPP is also important, which can be optimized by improving its downstream enzyme catalytic efficiency to “pull” the reaction rate from IPP/DMAPP to FPP and β-farnesene.

Therefore, the selection and introduction of efficient farnesene synthases is essential for constructing efficient farnesene producers using either yeast with the MVA pathway or *E. coli* and other prokaryotes with the MEP pathway. Farnesene synthase has been identified in various plants such as *Artemisia annua*, apple, *Mentha piperita*, *Picea abies*, soybean, and *Zea mays* (Tang et al., 2021; Liu et al., 2022a). Since the activities of farnesene synthases are different (Ye et al., 2022), it is necessary to screen farnesene synthases from different organisms to obtain those with high activities.

To date, the highest β-farnesene titer was 130 g/L achieved by recombinant *S. cerevisiae*, which was obtained through rewriting the central carbon metabolism to achieve commercial production (Meadows et al., 2016). By employing multiple engineering strategies and optimizing the fermentation conditions for the elevation of β-farnesene production in *Yarrowia lipolytica* with different substrates (Shi et al., 2021; Bi et al., 2022a; Liu et al., 2022b; Bi et al., 2023), a titer of 35.2 g/L farnesene was achieved with oleic acid as the feedstock (Liu et al., 2022b). Moreover, *Pichia pastoris* was also reported to produce 2.56 g/L farnesene (Liu et al., 2021). Compared to the high titer of β-farnesene achieved in yeast, the production in prokaryotes is relatively low. The highest farnesene titer was 10.31 g/L when utilizing the byproducts of crude glycerol in *E. coli* (Yao et al., 2020), but only milligram levels were reported in other prokaryotes such as *Synechococcus elongatus* PCC 7942, *Corynebacterium glutamicum*, and *Cupriavidus necator* H16 (Lee et al., 2017; Lim et al., 2020; Milker and Holtmann, 2021), which are far from the requirements for industrial applications. Farnesene production from different substrates in different strains of microorganisms is summarized in Supplementary Table S1. Hence, it is necessary to investigate the major obstacles affecting the development of prokaryotic chassis cells for efficient farnesene production.

*Zymomonas mobilis* is a facultative anaerobic bacterium that is generally regarded as safe (GRAS) and has excellent industrial characteristics, such as high sugar uptake and conversion efficiency, few byproducts, broad range of pH (pH 3.5–7.5) and temperature (24°C–40°C), phage resistance, mature genome-editing tools, and high-quality genome-scale metabolic model, which has made it an important chassis for constructing microbial cell factories for diverse biochemicals, such as lignocellulosic ethanol, PHB, lactate, acetoin, and isobutanol (Xia et al., 2019; Qiu et al., 2020; Li et al., 2022; Bao et al., 2023; Hu et al., 2023; Peng et al., 2024).

It is crucial to understand the metabolic and regulatory pathways underneath bacterial substance, energy, and information metabolisms to develop the microbial chassis cells into cell factories for the efficient production of target bioproducts. In *Z. mobilis*, glyceraldehyde 3-phosphate and pyruvate generated via the Entner–Doudoroff (ED) pathway are converted to FPP through the MEP pathway (Figure 1). Previous studies indicated that *Z. mobilis* is probably a suitable non-model microorganism to be developed for isoprenoid production. First, the ED pathway in *Z. mobilis* with fewer reactions than the Embden–Meyerhof–Parnas (EMP) pathway can facilitate a massive carbon flux from glucose to the MEP pathway. Second, the unique and highly thermodynamically favorable ED pathway generates high intracellular concentrations of pyruvate and glyceraldehyde 3-phosphate (Jacobson et al., 2019), which are important precursors of the MEP pathway. Finally, *Z. mobilis* can produce the unique and abundant membrane structures of hopanoids, which are also generated through the MEP pathway using FPP as the precursor (Martien et al., 2019; Khana et al., 2023), indicating the availability of FPP for farnesene biosynthesis in *Z. mobilis* (Figure 1).

In this study, β-farnesene synthases from various plant and fungus sources were selected and characterized with the β-farnesene-producing recombinant strains of *Z. mobilis* constructed, which was then optimized by employing systematic metabolic engineering strategies, including overexpressing β-farnesene synthase and increasing its copy numbers, balancing cofactor NADPH, and redirecting the carbon flux to the MEP pathway. Subsequently, β-farnesene fermentation conditions of C/N and aeration ratios were optimized to improve farnesene production in *Z. mobilis.*

## 2 Materials and methods

### 2.1 Strains, media, and culture conditions

*E. coli* DH5α and trans110 were used for routine cloning and plasmid demethylation, respectively. *E. coli* was cultured in lysogeny broth (LB) medium (10 g/L NaCl, 10 g/L tryptone, and 5 g/L yeast extract, pH 7.0) with shaking at 250 rpm in 37°C. The following antibiotics were used when required at the specified final concentrations: spectinomycin (100 μg/mL), kanamycin (50 μg/mL), and chloramphenicol (50 μg/mL).

*Z. mobilis* ZMNP, a native plasmid-free *Z. mobilis* ZM4 mutant strain (Geng et al., 2022), was used in this study. *Z. mobilis* strains were cultured in rich medium (RMG5; 50 g/L glucose, 10 g/L yeast extract, and 2 g/L KH_2_PO_4_, pH 5.8) with shaking at 100 rpm in 30°C. The following antibiotics were used when required at the specified final concentrations: chloramphenicol (50 μg/mL), spectinomycin (100 μg/mL), and kanamycin (200 μg/mL). Agar powder (1.5%, w/v) was added into the LB and RM broth for solid medium plate preparation. All strains used in this study are listed in Table 1.

**TABLE 1:** Strains used in this study.

Strains	Description	Source
DH5α	*E. coli* for plasmid construction	Lab stock
Trans110	*E. coli* for plasmid demethylation	Lab stock
ZMNP	Original strain	Geng et al. (2022)
FP0	ZMNP containing pEZ15A	This study
FP1	ZMNP containing pEZ15A_P*tet*-*ScBFS*	This study
FP2	ZMNP containing pEZ15A_P*tet-AaBFS*	This study
FP3	ZMNP containing pEZ15A_P*tet-MbBFS*	This study
FP4	ZMNP containing pEZ15A_P*tet-SmBFS*	This study
FP5	ZMNP containing pEZ15A_P*tet-ZmBFS*	This study
FP6	ZMNP containing pEZ15A_P*gap-AaBFS*	This study
FP7	ZMNP containing pEZ15A_*Pgap-AaBFS* and pEZ39p*_*P*gap-AaBFS*	This study
FP8	ZMNP with *ZMO1547* replaced by P*gap-AaBFS*	This study
FP9	ZMNP with *ZMO1547* and *ZMO1650* replaced by P*gap-AaBFS*	This study
FP10	FP6 containing pEZ39p*_*P*gap-zwf*	This study
FP11	FP6 containing pEZ39p*_*P*gap-ppnK*	This study
FP12	FP6 containing pEZ39p*_*P*gap-zwf*-*ppnK*	This study
FP13	FP10 containing pEZ15A_P*pdc*-*dxs*	This study
FP14	FP10 containing pEZ15A_P*pdc*-*dxs-ispG-ispH*	This study
FP15	ZMNP with pEZ15A_P*gap*-*AaBFS*_P*tet*-*idi* transferred	This study
FP16	ZMNP with *ZMO1547* replaced by P*gap*_*AaBFS*_P*tet*-*idi*	This study
FP17	FP10 with pEZ15A_P*pdc*-*dxs*-*dxr*	This study

### 2.2 DNA manipulation

Shuttle vectors pEZ15A (Yang et al., 2018) and pEZ39p (Li et al., 2022) were used in this study. The plasmid pEZ39p was constructed by replacing the origin of replication for *Z. mobilis* in pEZ15A with the gene-encoding replicase of the native plasmid pZM39 in *Z. mobilis* ZM4 and its native promoter.

The plasmid was constructed using the Gibson assembly method. The target genes were amplified using primers with a 15-bp nucleotide overlap to the adjacent DNA fragments. The fragments were separated by gel electrophoresis, purified using a Gel DNA Maxi Purification Kit (Tsingke, Wuhan, China), and subsequently quantified using a NanoDrop spectrophotometer (NanoDrop Technologies, DE, United States). The target fragment was mixed with the plasmid vector at a molar concentration ratio of 3: 1. Then, 0.5 U T5 exonuclease (NEB, MA, United States), 0.5 μL 10 × buffer, and Milli-Q ultrapure water were added to obtain a final volume of 5 μL. All the reagents were mixed and incubated on ice for 5 min. A total of 30 μL DH5α competent cells (Tsingke, Wuhan, China) were added to the 5-μL reaction system and left on ice for 15 min. The mixture was heated in a 42-C water bath for 45 s and then cooled on ice for 2 min. Resuscitation was performed by adding 200 μL LB medium and incubating at 250 rpm for 30 min at 37°C.

### 2.3 Construction of editing plasmids

Plasmid pL2R of the native type I-F CRISPR–Cas system (Zheng et al., 2019) was used to integrate the *AaBFS* gene into the genome at a chromosomal locus of *ZMO1547* and *ZMO1650* since their deletions do not affect the growth and ethanol production of *Z. mobilis* (Qiu et al., 2020; Xiao et al., 2024). The genome-editing plasmid was constructed according to the method described in the previous literature (Zheng et al., 2019). In brief, pL2R was digested with *Bsa*I at 37°C overnight. The spacer was a 32-bp sequence immediately after a 5′-CCC-3′ PAM, which was ordered from Tsingke Biotechnology Co., Ltd. (Tsingke, Wuhan, China). The oligonucleotides were mixed and heated to 95°C for 5 min and then annealed at room temperature. The digested linear DNA vector and the annealed spacer were enzymatically linked overnight by T4 ligase (Takara, Japan) at 18°C. The product after ligation was then transformed into *E. coli* DH5α competent cells. Donors were designed at the upstream and downstream of the knockout gene with a length of approximately 800 bp. Two donor fragments were connected to the target gene by overlapping PCR, and these fragments were amplified with primers with overlapping regions. All plasmids used in this study are listed in Table 2.

**TABLE 2:** Plasmids used in this study.

Name	Description	Source
pEZ15A	Shuttle vector containing *Z. mobilis* origin and *E. coli* origin 15A; Spe^R^	Yang et al. (2018)
pEZ39p	Shuttle vector containing *Z. mobilis* replicon	Li et al. (2022b)
pL2R	pEZ15A containing two artificial mini-CRISPR clusters based on the type I-F CRISPR–Cas system, Cm^R^	Geng et al. (2022)
pEZ15A_P*tet*-*AaBFS*	pEZ15A containing *AaBFS* driven by P*tet*	This study
pEZ15A_P*tet*-*MbBFS*	pEZ15A containing *MbBFS* driven by P*tet*	This study
pEZ15A_P*tet*-*ScBFS*	pEZ15A containing *ScBFS* driven by P*tet*	This study
pEZ15A_P*tet*-*SmBFS*	pEZ15A containing *SmBFS* driven by P*tet*	This study
pEZ15A_P*tet*-*ZmBFS*	pEZ15A containing *ZmBFS* driven by P*tet*	This study
pEZ15A_P*gap*-*AaBFS*	pEZ15A containing *AaBFS* driven by P*gap*	This study
pEZ39p*_*P*gap-zwf*	pEZ39p containing *ppnK* driven by P*gap*	This study
pEZ39p*_*P*gap-ppnK*	pEZ39p containing *zwf* driven by P*gap*	This study
pEZ39p*_*P*gap-zwf*-*ppnK*	pEZ39p containing genes *zwf* and *ppnK* driven by P*gap*	This study
pEZ39p*_*P*gap-AaBFS*	pEZ39p containing gene *AaBFS* driven by P*gap*	This study
pL2R_KOΔ*1547*	pL2R targeting *ZMO1547* containing the *ZMO1547* donor and *AaBFS* driven by P*gap*	This study
pL2R_KOΔ*1650*	pL2R targeting *ZMO1650* containing the *ZMO1650* donor and *AaBFS* driven by P*gap*	This study
pEZ15A_P*pdc*-*dxs*	pEZ15A containing *dxs* driven by P*pdc*	This study
pEZ15A_P*pdc*-*dxs-ispG-ispH*	pEZ15A containing *dxs ispG* and *ispH* driven by P*pdc*	This study
pEZ15A_P*gap*-*AaBFS*_P*tet*-*idi*	pEZ15A containing *AaBFS* under P*gap* and *idi* under P*tet*	This study
pL2R_KOΔ*1547_*P*gap*-*AaBFS*_P*tet*-*idi*	pL2R targeting *ZMO1547* and containing the *ZMO1547* donor and *AaBFS* under P*gap* and *idi* under P*tet*	This study
pEZ15A_P*pdc*-*dxs*-*dxr*	pEZ15A containing *dxs* and *dxr* driven by P*pdc*	This study

### 2.4 Recombinant strain screening

Recombinant plasmids were verified by Sanger sequencing (Tsingke, Wuhan, China) and then electroporated into the competent cells of *Z. mobilis* ZMNP using the Bio-Rad Gene Pulser (Bio-Rad, CA, United States). Immediately, the electroporated cells were transferred to 1 mL RMG5 and recovered at 30°C for 3∼6 h. Then, the cells were spread on RMG5 agar plates with appropriate antibiotics and incubated at 30°C for 2∼3 days. Corresponding primers were used to verify the target gene of the plasmid in the recombinant ZMNP strains, and the plasmid DNA was used as the positive control.

For genome editing, the validation primers were designed at a location of 100∼200 bp outside the donors, including *ZMO1547* and *ZMO1650*. The successfully edited strains were cultured in a liquid medium containing the corresponding antibiotics until the gene at this location was completely replaced by the target gene. Then, the recombinant strain was sub-cultured in RMG5 medium without any antibiotic until the editing plasmids were cured.

### 2.5 Shake-flask fermentation

For seed culture, the preserved bacterial glycerol stocks were cultured in 5 mL RMG5 liquid medium overnight and then transferred to RMG5 until the exponential phase. Then, the seed liquid was inoculated into a 50-mL flask containing 40 mL RMG5 at 30°C and 100 rpm with an initial OD_600nm_ value of 0.1. No dodecane was added for cell growth measurement.

Dodecane was added when analyzing β-farnesene production, and the ratio of RMG5 to dodecane was 5:1. Multiple samples were taken to analyze the relationship between β-farnesene accumulation and growth in recombinant strains. A measure of 2.5 mL medium was taken to analyze the glucose and ethanol content (Supplementary Figure S1). A measure of 500 μL dodecane was taken to analyze the β-farnesene yield (Supplementary Figure S2).

For fermentation condition optimization, the medium volumes in 100-mL flasks were 80 mL, 60 mL, and 40 mL. To prevent emulsification during culture due to too-low dodecane volume, the minimum medium volume was set to 40 mL. In addition, the volume of dodecane was optimized to 4 mL with 40 mL of RMG5 to maintain the ratio of 10:1 between the medium and dodecane. During fermentation processes with different carbon/nitrogen (C/N) ratios, 6 different ratios were utilized with the glucose and yeast extracts at 20:1, 10:1, and 5:1. In detail, the glucose and yeast extract ratios were performed at 50/2.5, 20/1, 50/5, 20/2, 50/10, and 20/4, respectively.

### 2.6 Analytical method

The ultraviolet visible spectrophotometer UV-1800 (Aoyi Instrument Co., Ltd., Shanghai, China) was used to detect the increase in the optical density of each strain at 600 nm. The concentration of glucose and ethanol was measured by high-performance liquid chromatography (HPLC) (Shimadzu, Tokyo, Japan) equipped with a refractive index detector (RID-20A) and a Phenomenex Rezex™ RFQ-Fast Acid H^+^ column (100 mm × 7.8 mm). The mobile phase (5 mM H_2_SO_4_) was set with a flow rate of 0.6 mL/min, column temperature of 80°C, and an injection volume of 2 μL.

The organic phase was filtered using a 0.2-μm syringe filter into HPLC vials. β-Farnesene concentrations were detected by HPLC (Shimadzu, Tokyo, Japan) equipped with an ultraviolet detector (SPD-20A) and a Shimadzu 5-μm C18 column (4.6 mm × 250 mm). The mobile phase with methyl alcohol:acetonitrile:water = 90:5:5 was set with a flow rate of 0.4 mL/min, column temperature of 30°C, and an injection volume at 2 μL, as previously described.

The data presented in the graphs were analyzed with the mean ± SD by utilizing GraphPad Prism software (version 9.0.0) to calculate the mean standard deviation and perform *t*-tests or one-way ANOVA. *p* < 0.05 was considered as a statistically significant difference.

## 3 Results and discussion

### 3.1 Screening and selection of effective β-farnesene synthases

The crucial step for β-farnesene production *in vivo* is to maximize the carbon flux from FPP to β-farnesene, which is catalyzed by FSs (Tang et al., 2021) (Figure 1). Until now, the identified FS usually comes from various plants, and only a few farnesene synthases have been identified in fungi through gene horizontal transfer (Jia et al., 2019), which include *Streptomyces coelicolor* (Lin et al., 2006), *A. annua* (You et al., 2017), *Metarhizium brunneum*, *Salvia miltiorrhiza*, and *Z. mays* (Köllner et al., 2009).

**FIGURE 2:**
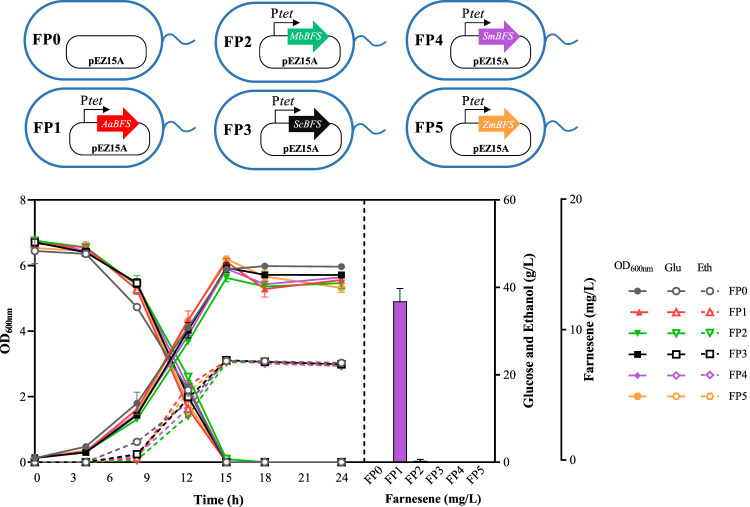
Cell growth of OD_600nm_, glucose consumption, ethanol production, and farnesene titer of different recombinant strains of *Z. mobilis*. FP0 containing a blank pEZ15A plasmid was used as the control. The remaining strains of FP1, FP2, FP3, FP4, and FP5 all contain a pEZ15A plasmid with farnesene synthases from different organisms driven by the inducible promoter of P*tet*. Each experiment was performed with at least three replicates. The inducer concentration of the tetracycline was 0.8 μg/mL.

Because the characteristics of FSs obtained from different species vary greatly, genes encoding five β-farnesene synthases from *A. annua*, *M. brunneum*, *S. coelicolor*, *S. miltiorrhiza*, and *Z. mays* (Table 3) were selected with their β-farnesene production potential, as specified in previous studies (Picaud et al., 2005; Lin et al., 2006; Köllner et al., 2009). All of these five farnesene synthases were codon-optimized for *Z. mobilis* first. Then, five plasmids were constructed using the shuttle vector pEZ15A with FS genes driven by the inducible promoter P*tet*, which were then transformed into ZMNP to generate recombinant strains of FP1, FP2, FP3, FP4, and FP5 containing *AaBFS*, *MbBFS*, *ScBFS*, *SmBFS*, and *ZmBFS*, respectively. Only FP1 and FP2 strains in RMG5 had farnesene that was detected with a titer of 12.40 ± 1.00 mg/L and 0.20 ± 0.01 mg/L, respectively (Figure 2). The result that FP1-containing *AaBFS* from *A. annua* had the highest β-farnesene production in *Z. mobilis* is consistent with that of previous works using other microorganisms such as *S. cerevisiae, Y. lipolytica*, and *E. coli* (Meadows et al., 2016; You et al., 2017; Shi et al., 2021; Bi et al., 2023), which also suggests that *AaBFS* could be a suitable FS for β-farnesene biosynthesis in other microorganisms. However, it is worthy of further exploration of other farnesene synthases with the explosive accumulation of microbial genome sequences and the rapid development of novel protein design tools.

**TABLE 3:** β-Farnesene synthase selected from different organisms.

Enzyme	Organism	Length (aa)
AaBFS	*Artemisia annua*	574
MbBFS	*Metarhizium brunneum*	331
ScBFS	*Streptomyces coelicolor*	461
SmBFS	*Salvia miltiorrhiza*	589
ZmBFS	*Zea mays*	554

### 3.2 Enhancement of the expression of β-farnesene synthase to increase farnesene production

**FIGURE 3:**
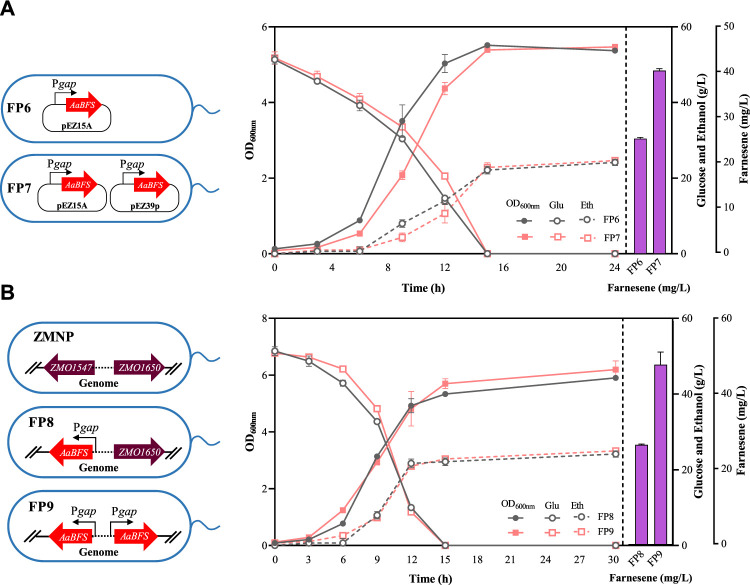
Cell growth of OD_600nm_, glucose consumption, ethanol production, and farnesene titer in the recombinant strains of FP6 and FP7 **(A)** and FP8 and FP9 **(B)**. Each experiment was performed with at least three replicates.

To further increase β-farnesene production, the inducible promoter P*tet* utilized in FP1 was replaced with a strong constitutive promoter P*gap* in *Z. mobilis* to generate the recombinant strain FP6, which can produce 25.73 ± 0.31 mg/L β-farnesene, which is a more than 2-fold increase compared to that in FP1 (Figure 3A), which is also consistent with the results of changing promoter strength to improve farnesene production in other microorganisms, such as P*trc* in *E. coli*, P_*T7*_ in *S. cerevisiae*, and P_*TEF*_ in *Y. lipolytica* (Wang et al., 2011; Zhu et al., 2014; Bi et al., 2022b).

Another classical strategy to enhance gene expression is to increase its copy numbers. Therefore, another shuttle plasmid pEZ39p containing another copy of P*gap*–*AaBFS* was constructed and then transformed into FP6 to generate FP7. The β-farnesene production of FP7 achieved 41.00 ± 0.40 mg/L (Figure 3A), which was a more than 59% increase over its parental strain FP6 carrying only one plasmid.

Considering the instability of plasmids, especially the co-existence of two plasmids containing the same construct of P*gap*–*AaBFS* and the potential plasmid incompatibility between two plasmids, the copy number effect in chromosomes was also investigated. First, the P*gap*–*AaBFS* construct of β-farnesene synthase driven by P*gap* was used to replace the *ZMO1547* gene in the ZMNP genome to generate FP8. The β-farnesene titer of FP8 with one copy of the P*gap*–*AaBFS* construct was 26.80 ± 0.20 mg/L, which was similar to that of FP6 containing a copy of P*gap*–*AaBFS* in the plasmid (Figure 3A). The copy number of FSs was increased by genome multi-locus integrations with the *ZMO1650* gene in the FP8 genome replaced by another copy of the P*gap*–*AaBFS* construct to generate FP9. Similarly, FP9 containing two copies of P*gap*–*AaBFS* in the genome also increased the β-farnesene titer to 48.33 ± 3.40 mg/L, a more than 80% increase compared with that of FP8 (Figure 3B) and an 18% increase compared to that of FP7 containing two plasmid copies (Figure 3A). Furthermore, we increased the third *AaBFS* copy number using the plasmid pEZ33p in strain FP7 to help promote the β-farnesene titer, which was named FP15. However, the β-farnesene production in FP15 was 18.47 ± 1.10 mg/L, which was significantly decreased compared with that of FP7 (Supplementary Figure S3).

Previous studies reported that the β-farnesene titer usually increased with the increase in the copy number (Shi et al., 2021; Ye et al., 2022). For example, the recombinant *S. cerevisiae* strain JVA139 with a copy number of 5 achieved the highest β-farnesene titer (Ye et al., 2022). However, the applicability of the augment of the copy number of FSs in microorganisms varies between species. For example, there was little improvement in β-farnesene production when the BFS copy number increased to 4 in *Y. lipolytica* CIBT6293 (Shi et al., 2021).

### 3.3 NADPH cofactor balancing

The redox state of cells profoundly impacted gene expression and global metabolism. NADPH and NADH cofactors usually serve as the essential reducing factors in biosynthesis processes, and the availability of NADPH contributes to farnesene production (Mookhtiar et al., 1994; Liu et al., 2014; Chen et al., 2023). In prokaryotes, the important enzymes of 1-deoxy-D-xylulose-5phosphate reductoisomerase (DXR), 1-hydroxy-2-methyl-2-butenyl-4-diphosphate synthase (IspG), and 1-hydroxy-2-methyl-2-butenyl-4-diphosphate reductase (IspH) in the MEP pathway are ferredoxin-dependent reductases (TAKAHASHI et al., 1998; Frank and Groll, 2016). Cofactor balancing of NADPH is widely applied in many microorganisms for optimal biochemical production (Chen et al., 2023; Fan et al., 2024). Previously, the introduction of the ED pathway from *Z. mobilis* into *E. coli* MG1655 increased the rate of NADPH regeneration by 25-fold and, thus, improved terpenoid production (Ng et al., 2015), and the effectiveness of NADPH generation by overexpressing Zwf (ZMO0367) and PpnK (ZMO1329) to promote NADPH-dependent metabolisms was also demonstrated in *Z. mobilis* (Li et al., 2022).

**FIGURE 4:**
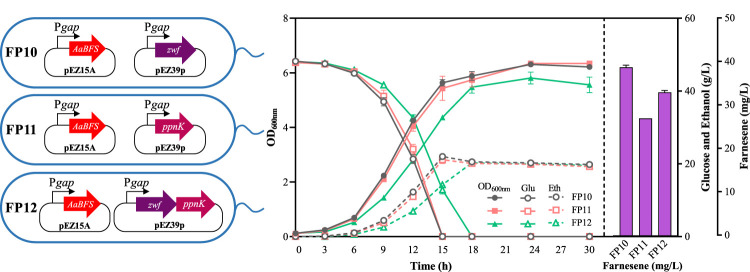
Cell growth of OD_600nm_, glucose consumption, ethanol production, and farnesene titer of the recombinant strains FP10, FP11, and FP12. Each experiment was performed with at least three replicates.

Zwf and PpnK were then overexpressed to balance the reducing equivalents for β-farnesene biosynthesis in *Z. mobilis*. *Zwf* or *ppnK* genes controlled by a strong constitutive promoter P*gap* were electroporated into FP6 alone or together to generate recombinant strains of FP10, FP11, and FP12 (Figure 4). Compared with FP6, cell growth, glucose consumption, and ethanol production of these three strains were reduced, especially in strain FP12 (Figure 4). The β-farnesene titer of FP10 was increased to 39.07 ± 0.50 mg/L. It should be noted that no significant promotion of β-farnesene was found in both FP11 and FP12 with the overexpression of *ppnK* alone or in combination with *zwf*. β-Farnesene biosynthesis requires the supply of NADPH as a cofactor. Overexpressing *zwf* in *Z. mobilis* can provide NADPH directly, thus generating a higher β-farnesene titer in FP10. However, PpnK catalyzes the conversion of NAD^+^ to NADP^+^. Its overexpression in FP11 only provides NADP^+^, which needs further conversion to generate the required NADPH. The co-overexpression of *zwf* and *ppnK* in FP12 may cause an imbalance of reducing power for *Z. mobilis.* Therefore, the balance of the reducing power is an important cell energetic requirement for efficient metabolism and biochemical production.

### 3.4 Optimization of the MEP pathway to redirect the carbon flux for farnesene production

For the major objective of high farnesene production, it is crucial to divert sufficient carbon flux to the pathway for farnesene biosynthesis while maintaining normal cell growth. Therefore, it is viable to promote the farnesene titer through metabolic pathway modification to obtain an adequate metabolic flux supply. Previous studies in various microbes including *E. coli*, *Bacillus subtilis*, *C. glutamicum*, and *Rhodobacter sphaeroides* demonstrated that the overexpression of the MEP pathway enzymes is effective in promoting their pathway activity (Zhu et al., 2014; Tang et al., 2021; Liu et al., 2022a).

**FIGURE 5:**
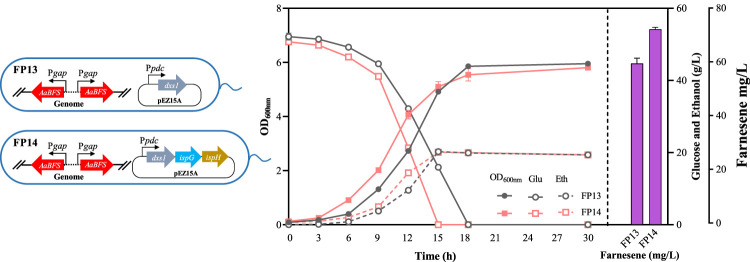
Cell growth of OD_600nm_, glucose consumption, ethanol production, and farnesene titer of the recombinant strains of FP13 and FP14. Each experiment was performed with at least three replicates.

In this study, the gene encoding 1-deoxyD-xylulose-5-phosphate synthase (DXS) of the MEP pathway was first overexpressed in FP9 to generate FP13 (Figure 5). The introduction of *dxs* increased the β-farnesene titer to 60.47 ± 1.96 mg/L in FP13. Considering that the function of IspG and IspH relies on the dioxygen-sensitive iron–sulfur [4Fe–4S] cluster during the reduction process with a redox shuttle, such as flavodoxin/flavodoxin reductase/NADPH (Wang and Oldfield, 2014; Li et al., 2018), three genes encoding DXS, IspG, and IspH were also overexpressed, driven by a strong constitutive promoter P*pdc* in FP9 to obtain FP14 (Figure 5). As a result, the growth of FP14 was significantly faster than that of FP13 (Figure 5), which may be ascribed to the “push” strategy of diverting 2-methyl-erythritol 2, 4 cyclodiphosphate (MEcDP) to downstream metabolites of 1-hydroxy-2-methyl-2-butenyl 4-diphosphate (HMBDP), IPP, and DMADP for farnesene synthesis to reduce the toxic effects on *Z. mobilis*. After overexpressing the three genes of *dxs*, *ispG*, and *ispH* in FP14, the final β-farnesene titer reached 73.30 ± 0.70 mg/L, which was 1.52-fold more than that in FP9 (Figure 5).

DXS has been demonstrated to be a pivotal rate-limiting enzymatic bottleneck in the MEP pathway, which has been widely used in various microbes, including *E. coli*, *B. subtilis*, and *C. glutamicum* (Xue and Ahring, 2011; Lv et al., 2012; Liu et al., 2014; Li et al., 2018; Lim et al., 2020). Its abundance usually serves as a key point in the MEP pathway activity regulation in *Z. mobilis* (Martien et al., 2021). More importantly, the protein level increase of DXS2 in *Z. mobilis* is also beneficial for remarkable increases of all MEP pathway intermediates, especially the intracellular levels of 1-deoxy-d-xylulose 5-phosphate (DXP), 4-diphosphocytidyl-2-methyl-erythritol (CDP-ME), and MEcDP (Khana et al., 2023). Despite the observed increase in β-farnesene production after DXS overexpression in FP13, it significantly affected strain cell growth, suggesting that the cellular accumulated intermediates may be toxic to *Z. mobilis* cells. A similar result was also reported where the buildup of MEcDP only caused relatively minor increases in HMBDP and IPP/DMADP as the downstream MEP metabolites (Khana et al., 2023). The cellular metabolic flux in FP13 may not convert MEcDP into its downstream intermediates efficiently. This might be the main reason for the reduced growth of FP13, although the mechanism of its toxicity needs further investigation.

Therefore, it is of great importance to optimize the expression and activity of the downstream enzymes. As the last two key enzymes of the MEP pathway, IspG and IspH are also considered to be the two other restrictive points (Li et al., 2018). A previous study demonstrated that the overexpression of *ispG* can reduce MEcDP efflux by possibly converting it directly to its downstream products in *E. coli* (Appanna et al., 2012). However, the accumulation of intermediate HMBPP after *ispG* overexpression may interfere with protein and nucleotide synthesis in *E. coli*. In addition, the lower limonene productivity after the overexpression of *ispG* alone in the *S. elongatus* strain L1118 also indicated its limited role in enhancing MEP flux (Li et al., 2022). The problem of HMBPP accumulation can also be addressed by further activating its downstream enzyme IspH to divert the carbon flux away from MEcDP and to help promote β-carotene and lycopene production (Li et al., 2017). In addition, the overexpression of *ispG* with *dxs* also led to a 3.3-fold increase in isopentenol production in *E. coli* (Liu et al., 2014). Similarly, a systematic analysis of the MEP pathway intermediate metabolites in *Z. mobilis* also indicated that the overexpression of *dxs*, *ispG*, and *ispH* can mitigate MEcDP to downstream intermediates (Khana et al., 2023). The improved cell growth combined with promoted β-farnesene production in FP14 demonstrated the effectiveness of mitigating metabolic bottlenecks by overexpressing IspG and IspH to increase the IPP/DMAPP pool for improved farnesene production.

Overexpression of DXR and isopentenyl diphosphate isomerase (IDI) has also been conducted. However, no significant promotion of β-farnesene production was detected in the generated strains. This result is similar with a previous result that only a modest decrease in DXP levels (0.46-fold) was detected, and no significant changes were found in the downstream intermediates levels (Khana et al., 2023). Therefore, DXR was recognized as a less important enzymatic constraint in the *Z. mobilis* MEP pathway. It was previously reported that after individual overexpression of methylerythritol phosphate cytidylyltransferase (IspDF), the levels of CDP-ME and MEP were promoted by 78-fold and 3.8-fold, respectively (Khana et al., 2023). In contrast, the farnesene titer was not increased in this study after the application of the same strategy (data not shown). Despite the rationale for this result remaining unclear, it is plausible that the allosteric regulation of these enzymes may exert an influence. Furthermore, enzymes of IspD and IspF appear to be minor bottlenecks, and they are often ignored by the studies of metabolic engineering for isoprenoid production (Li et al., 2018). Overall, the major goal for MEP pathway metabolic engineering is to balance and regulate the expression levels of multiple genes to prevent intermediate accumulation and push the metabolic flux toward the desired direction. However, although some MEP intermediate levels can be increased after overexpressing the relevant enzymes, they progressively diminished over time following their initial surge (Khana et al., 2023), which also indicated the ineffectiveness of overexpressing *dxr*, *idi,* and *ispDF*.

### 3.5 Optimization of fermentation conditions

C/N ratios of the culture medium affected microbial cell growth and, consequently, the biochemical production. For example, the recombinant *Y. lipolytica* strain had the highest farnesene titer with a C/N ratio of 15 compared with the C/N ratios of 10 and 20 (Bi et al., 2023), and the C/N ratios and the amount of carbon and nitrogen affected the PHB accumulation in the recombinant *Z. mobilis* strain (Li et al., 2022). β-Farnesene accumulation under different ratios of C/N was also investigated in this study with an 80% flask medium volume and a medium/dodecane ratio of 5:1.

**FIGURE 6:**
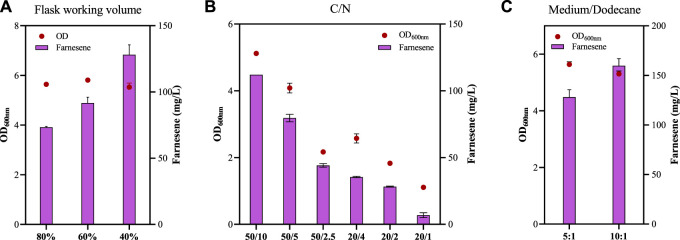
Cell growth of OD_600nm_ and farnesene titer of the recombinant strain FP14 under different C/N ratios **(A)**, flask working volumes **(B)**, and medium/dodecane ratios **(C)**. Each experiment was performed with at least three replicates.

*Z. mobilis* is a facultative anaerobic industrial microorganism with advantages in economic biochemical production under anaerobic conditions, and the fermentation condition of the 80% flask working volume used above is considered an anaerobic condition relatively. The effect of different flask working volumes of 80%, 60%, and 40% on farnesene production was further investigated (Figure 6A). No significant difference of OD_600nm_ in the FP14 strain was observed under conditions of flask working volumes of 80%, 60%, and 40%. However, β-farnesene production increased significantly from 73.30 ± 0.71 mg/L in the 80% working volume to 91.67 ± 4.41 mg/L in the 60% working volume and 128.10 ± 7.50 mg/L in the 40% working volume (Figure 6A). This result is consistent with that of a previous study, which stated that the exposure of *Z. mobilis* to oxygen profoundly influenced its transient and long-term metabolism, including the MEP pathway, leading to the accumulation of the metabolic intermediate MEcDP in this pathway (Martien et al., 2019), which may be the reason that a 40% flask working volume is preferred for farnesene production in *Z. mobilis*.

The amount of carbon sources and the C/N ratios affected farnesene production, while the amount of nitrogen may be the major factor for cell growth (Figure 6B). Cell growth and farnesene production decreased with the increase in C/N ratios from 5/1 to 10/1 and then 20/1, regardless of the initial glucose concentration of 20 g/L or 50 g/L (Figure 6B). For example, OD_600nm_ values of FP14 at the C/N ratios of 50/10, 50/5, and 50/2.5 were 5.17, 4.08, and 2.17, respectively. The corresponding farnesene titers at the C/N ratios of 50/10, 50/5, and 50/2.5 were 112.00 ± 0.00 mg/L, 79.60 ± 2.80 mg/L, and 44.13 ± 1.40 mg/L, respectively (Figure 6B).

Finally, the impact of the medium/dodecane ratios was analyzed. When the ratio of the medium/dodecane changed from 5/1 and 10/1, OD_600nm_ of FP14 decreased slightly from 5.64 to 5.30, while the β-farnesene titer increased significantly from 128.10 ± 7.50 mg/L to 159.70 ± 7.21 mg/L (Figure 6C). The reduced dodecane in the medium provides more opportunities for the strains to make contact with oxygen, thus helping to promote β-farnesene production by 24.67%. In summary, after systematic metabolic engineering and fermentation optimization, the final β-farnesene production in FP14 was improved by nearly 13-fold more than that in the parental strain FP1 in shake flasks. In spite of this, farnesene production in *Z. mobilis* was still not comparable to that of the other strains shown in Supplementary Table S1. In the future, more strategies will be developed to further enhance β-farnesene production. First, considering the importance of farnesene synthase, its catalytic activity can be promoted through rational or irrational designs. Second, the provision of reducing power and ATP will also benefit its production. Third, the competitive carbon flux of its native ethanol pathway can be redirected to farnesene synthesis through model-guided metabolic pathway optimization.

## 4 Conclusion

**FIGURE 7:**
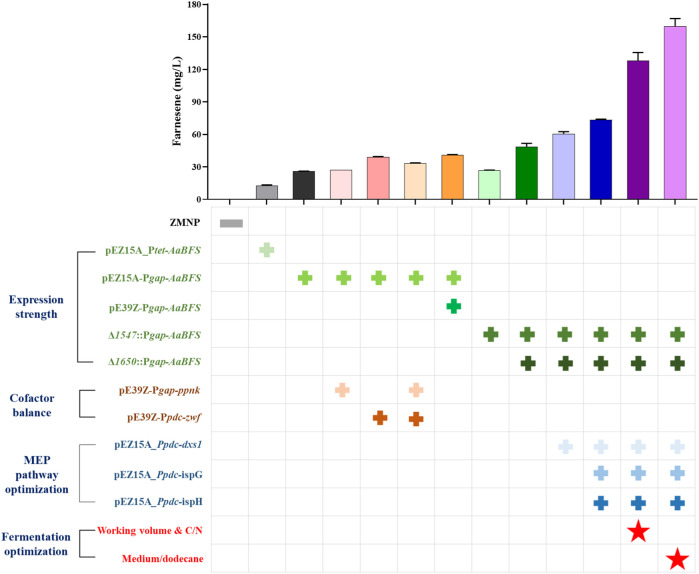
Summary of β-farnesene accumulation in recombinant *Z. mobilis* strains with systematic metabolic engineering strategies. Different strategies of expression strength, cofactor balance, and MEP pathway optimization are indicated by the symbol “+” in green, brown, and blue, respectively. The strategy of fermentation optimization was noted with “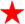.”

In conclusion, systematic metabolic engineering strategies were employed to create β-farnesene production recombinant strains in *Z. mobilis*, a non-model facultative anaerobic ethanologenic bacterium with excellent industrial characteristics. A heterologous β-farnesene synthase *AaBFS* was selected from the five β-farnesene synthases screened, and the recombinant strain FP1 containing *AaBFS* driven by an inducible promoter P*tet* produced 12.40 ± 1.00 mg/L β-farnesene. The expression of *AaBFS* was further enhanced through the strategies of using a strong constitutive promoter P*gap* and increasing the gene copy numbers. The recombinant strain FP6 containing *AaBFS* driven by P*gap* (P*gap*–*AaBFS*) doubled the β-farnesene production to 25.73 ± 0.31 mg/L. The recombinant strains of FP7 and FP9 containing two copies of P*gap*–*AaBFS* in plasmids or different genomic loci had β-farnesene titers further increased to 41.00 ± 0.40 and 48.33 ± 3.40 mg/L, respectively. The recombinant strain FP14 generated from FP9 with carbon flux redirected to the MEP pathway by overexpressing the genes *dxs*, *ispG*, and *ispH* encoding the first and last two enzymes of the MEP pathway can produce β-farnesene at a titer of 128.10 ± 7.50 mg/L. Finally, the highest β-farnesene production of 159.70 ± 7.21 mg/L was obtained after optimizing the fermentation conditions, including the ratios of C/N, flask working volume, and medium/dodecane in FP14. All the strategies used in this study and the corresponding β-farnesene titers are shown in Figure 7. This work, thus, not only generated a recombinant *Z. mobilis* strain with the highest β-farnesene production reported so far in flask fermentation but also unraveled the bottlenecks to engineer *Z. mobilis* for farnesene production, which could help guide future rational design and the construction of microbial cell factories for terpenoid production in non-model industrial microorganisms such as *Z. mobilis*.

## Data Availability

The original contributions presented in the study are included in the article/Supplementary Material; further inquiries can be directed to the corresponding authors.
