# Supplementary material for: Systematic metabolic engineering of Zymomonas mobilis for β-farnesene production
Source: Front Bioeng Biotechnol. 2024 May 17;12:1392556. doi: 10.3389/fbioe.2024.1392556 (PMC11140730; doi:10.3389/fbioe.2024.1392556)
Supplement: Supplementary file 1 [file DataSheet1.docx]

**Systematic Metabolic Engineering** **of *Zymomonas mobilis***

**for β-farnesene Production**

Yubei Xiao, Xuemei Tan, Qiaoning He^*^, Shihui Yang^*^

State Key Laboratory of Biocatalysis and Enzyme Engineering, and School of Life Sciences, Hubei University, Wuhan, 430062, China

**Email:**

YX: xyb@hubu.edu.cn

XT: XuemeiTan@stu.hubu.edu.cn

QH: Qiaoninghe@hubu.edu.cn

SY: Shihui.Yang@hubu.edu.cn

∗ Corresponding author.

**Table S1. Farnesene titer of existing literatures.**

| **Strains** | **Substrates** | **Titer** | **Fermentation time** | **Reference** |
| --- | --- | --- | --- | --- |
| *S. cerevisiae* | Cane syrup | Over 100 g/L | 6-day fed-batch | (Meadows et al., 2016) |
| *Y. lipolytica* | Glucose | 22.8 g/L | 240 h fed-batch | (Shi et al., 2021) |
| *Y. lipolytica* | Hydrolysate | 7.38± 0.24 g/L | 144 h fed-batch | (Bi et al., 2022) |
| *Y. lipolytica* | Oleic acid | 3.34 g/L | 96 h shake-flask | (Liu et al., 2022) |
| *Y. lipolytica* | Glucose | 810 mg/L | 96 h shake-flask | (Bi et al., 2023) |
| *P. pastoris* | Oleic acid/sorbitol | 2.56 g/L | 72 h shake-flask | (Liu et al., 2021) |
| *E. coli* | Crude glycerol | 10.31g/L | Fed-batch | (Yao et al., 2020) |
| *E. coli* | Biodiesel | 3.31 g/L | 96 h shake-flask | (You et al., 2017) |
| *E. coli* | Whey powder | 2.41 g/L | 48 h shake-flask | (Ding et al., 2021) |
| *C. necator* | Fructose | 5.4 ± 0.3 mg/L | Fed-batch | (Milker and Holtmann, 2021) |

**Table S2.** **The amino acid sequences of farnesene synthases from different organisms.**

**>AaBFS:** MSTLPISSVSFSSSTSPLVVDDKVSTKPDVIRHTMNFNASIWGDQFLTYDEPEDLVMKKQLVEELKEEVKKELITIKGSNEPMQHVKLIELIDAVQRLGIAYHFEEEIEEALQHIHVTYGEQWVDKENLQSISLWFRLLRQQGFNVSSGVFKDFMDEKGKFKESLCNDAQGILALYEAAFMRVEDETILDNALEFTKVHLDIIAKDPSCDSSLRTQIHQALKQPLRRRLARIEALHYMPIYQQETSHDEVLLKLAKLDFSVLQSMHKKELSHICKWWKDLDLQNKLPYVRDRVVEGYFWILSIYYEPQHARTRMFLMKTCMWLVVLDDTFDNYGTYEELEIFTQAVERWSISCLDMLPEYMKLIYQELVNLHVEMEESLEKEGKTYQIHYVKEMAKELVRNYLVEARWLKEGYMPTLEEYMSVSMVTGTYGLMIARSYVGRGDIVTEDTFKWVSSYPPIIKASCVIVRLMDDIVSHKEEQERGHVASSIECYSKESGASEEEACEYISRKVEDAWKVINRESLRPTAVPFPLLMPAINLARMCEVLYSVNDGFTHAEGDMKSYMKSFFVHPMVV*

**>MbBFS:** METDSFKRQYADILRRYLCGISYQKLSCEYDPSIEETVVQHFRTLNFPNDFLKRMMPIIHASAWIATSTYPFTPRHVQEAIAVYTSLAIAIEDTSKESTHDLKRFQQRLFNRQPQPNLLLQAMVDCLVSLRGIYGPFICDMVAKSTAEYISVCAFEAKYDGTLRPTPSSPDFPYYLRLKTGVAEVYAFFAFPEVLYPEEAFLHEYILAVPDISRYFNLGNDLLSFYKESIVADERLNYIYNCSRVSNSTPLESIWSTHLALITCVENIRKTLSASPQMRRNIDQLINGYVMYHFGASRYKLSDLGIQEVDELRAKICCSTTVDNGVGAYKH*****

**>ScBFS:** MTVESVNPETRAPAAPGAPELREPPVAGGGVPLLGHGWRLARDPLAFMSQLRDHGDVVRIKLGPKTVYAVTNPELTGALALNPDYHIAGPLWESLEGLLGKEGVATANGPLHRRQRRTIQPAFRLDAIPAYGPIMEEEAHALTERWQPGKTVDATSESFRVAVRVAARCLLRGQYMDERAERLCVALATVFRGMYRRMVVPLGPLYRLPLPANRRFNDALADLHLLVDEIIAERRASGQKPDDLLTALLEAKDDNGDPIGEQEIHDQVVAILTPGSETIASTIMWLLQALADHPEHADRIRDEVEAVTGGRPVAFEDVRKLRHTGNVIVEAMRLRPAVWVLTRRAVAESELGGYRIPAGADIIYSPYAIQRDPKSYDDNLEFDPDRWLPERAANVPKYAMKPFSAGKRKCPSDHFSMAQLTLITAALATKYRFEQVAGSNDAVRVGITLRPHDLLVRPVAR*

**>SmBFS:** MSTTSLRTPILSMPEIHLHKLERRASKAYGVCCTARARLQVSCSSPLQVRRSGNYKPSLWDFTHIQSLNTHYKEESHLNREAELILQVKMLLQDKMEAVEQLELIHDFKYLGLSYFFQQEIKQILSFIYITHACFHDNKTEEKDLYFTALGFRILRQHGFNVSQEVFDCFKNERGSDFKESLAQDTKGMLQLYEASFLLREGEDTLELARQFSTKCLQRKLEEGGDEIDINLISWIRHSLEIPLHWRTQNLEARWFLDAYARRPDMNPIVFELAKLDFNIVQATQQQELKDISRWWNNSCLAEKLPFVRDRLVESYFWAIALFESHENGYHRKTAAKIITLITALDDVYDIYGTLDELELFTDAIRRWDTESMNRLPYYMQLFYFVIYNLVSEMAYDILKEQGFISIPYLQKSWVNLVEAYLQEAKWYYSGYIPSMEEYLNNSMISIGAPTVISQVFFTLGTSKGKPVVESFFKYNHIFRLSGMLVRLPDDLGTSRFEMKRGDVAKSIQCYMKERNGSEKEAEEHVRFMIREVWKEMNTATATAAAPHDLVETAANLGRAAQFMYLDGDGNHSKLHQRIASLLFHPYLL*

**>ZmBFS:** MASPPAHRSSKAADEELPKASSTFHPSLWGSFFLTYQPPTAPQRANMKERAEVLRERVRKVLKGSTTDQLPETVNLILTLQRLGLGYYYENEIDKLLHQIYSNSDYNEKDLNLVSQRFYLLRKNGYDVPSDVFLNFKTEEGGFACAAADTRSLLSLYNAAYLRKHGEEVLDEAISSTRLRLQDLLGRLLPESPFAKEVSSSLRTPLFRRVGILEARNYIPIYEKEATRNEAVLELAKLNFNLQQLDFCEELKHCSAWWNEMIAKSKLTFVRDRIVEEYFWMNGACCDPPYSLSRIILTKITGLITIIDDMFDTHGTTEDCMKFAEAFGRWDESAIHLLPEYMKDFYILMLETFQSFEDALGPEKSYRVLYLKQAMERLVELYSKEIKWRDQDYVATMSEHLQVSAESIGANALTCSAYAGMGDMSITKETFEWALSFPQFIRTFGSFVRLSNDVVSTKREQTKDHSPSTVHCYMKEHGTTMDDACEKIKELIEDSWKDMLEQSLALKGLPKVVPQLVFDFSRTTDNMYRDRDALTSSEALKEMIQLLFVEPIPE*

**Table S3. Plasmids used in supplementary material.**

| **Name** | **Description** | **Sources** |
| --- | --- | --- |
| pEZ33p | ZMOp33×028 and *E. coli* origin 15A, Cm^R^ | (Yang et al., 2018) |
| pEZ33p-_P*gap*-*AaBFS* | pEZ33p contains gene *AaBFS* under P*gap* | This study |
| pEZ15A_P*gap*-*AaBFS*_P*tet*-*idi* | Plasmid pEZ15A contains gene *AaBFS* under P*gap* promoter and *idi* under P*tet* promoter | This study |
| pL2R_KOΔ*1547_*P*gap*-*AaBFS*_P*tet*-*idi* | Plasmid pL2R targets *ZMO1547* and contains *ZMO1547* donor and gene *AaBFS* under P*gap* promoter and gene *idi* under P*tet* promoter | This study |
| pEZ15A_P*pdc*-*dxs1*-*dxr* | Plasmid pEZ15A contains gene *dxs1* and *dxr* under P*pdc* promoter | This study |

**Table S4.** **Strains used in supplementary material.**

| **Strains** | **Description** | **Sources** |
| --- | --- | --- |
| FP15 | FP7 with pEZ33p_P*gap*-*AaBFS* transferred | This study |
| FP16 | ZMNP with pEZ15A_P*gap*-*AaBFS*_P*tet*-*idi* transferred | This study |
| FP17 | ZMNP with *ZMO1547* knockout and replaced by P*gap_AaBFS_*P*tet-idi* | This study |
| FP18 | FP10 with pEZ15A_P*pdc*-*dxs1*-*dxr* transferred | This study |

**
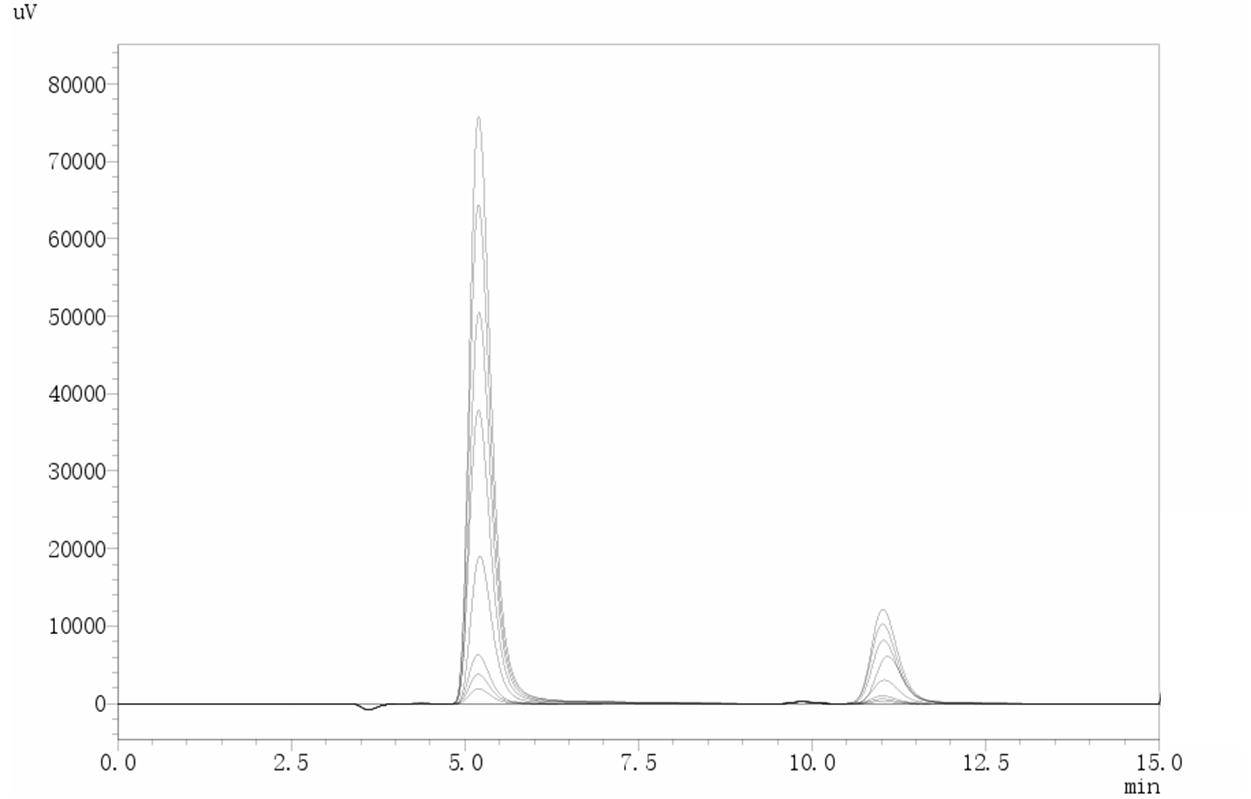
**

**Figure S1. The overlapping peaks of glucose and ethanol standards.** The concentrations of glucose were 1.5, 3, 5, 15, 30, 40, 50, and 60 g/L, respectively. The concentrations of glucose were 0.75, 1.5, 2.5, 7.5, 15, 20, 25, and 30 g/L, respectively. And the R^2^ was 0.999, indicating high reliability of this data. The peak times of glucose and ethanol were about 5.19 min and 10.97 min, respectively.

**
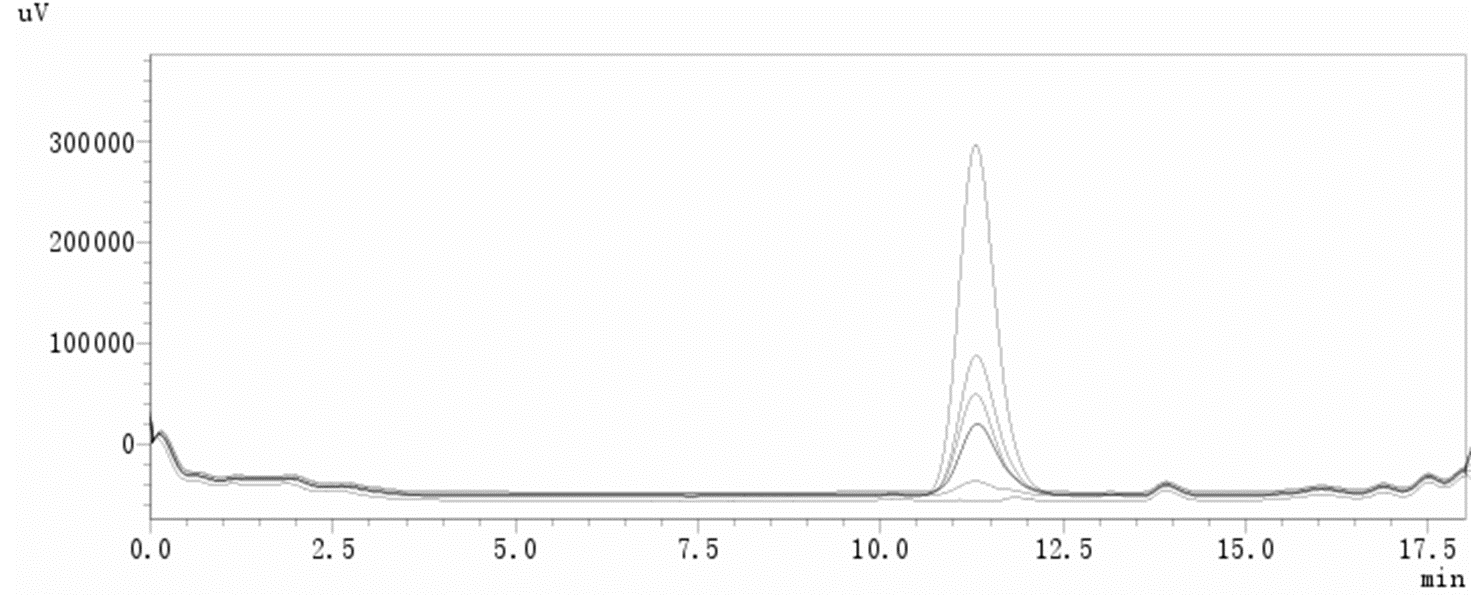
**

**Figure S2. The overlapping peak of β-farnesene standard.** The β-farnesene concentrations were 0.02, 0.05, 0.1, 0.2, 0.5, and 1 g/L, respectively. And the R^2^ was 0.999, indicating high reliability of this data. The peak time was about 11.22 min.

**Figure S3. The schematic diagram of farnesene synthase selection and cell growth of OD_600nm_, glucose consumption, ethanol production, and farnesene titer of FP15.** Each experiment was performed with at least three replicates.

**F****igure S4. Cell growth of OD_600nm_, glucose consumption, ethanol production, and farnesene titer in recombinant strains of FP16 and FP17.** Each experiment was performed at least three replicates.

**Figure S5. Cell growth of OD_600nm_, glucose consumption, ethanol production, and farnesene titer in recombinant strain FP18.** Each experiment was performed at least three replicates.

**Reference**

Bi, H., Xu, C., Bao, Y., Zhang, C., Wang, K., Zhang, Y., et al. (2023). Enhancing precursor supply and modulating metabolism to achieve high-level production of β-farnesene in *Yarrowia lipolytica*. *Bioresour. Technol.* 382. doi: 10.1016/j.biortech.2023.129171.

Bi, H., Xv, C., Su, C., Feng, P., Zhang, C., Wang, M., et al. (2022). β-Farnesene production from low-cost glucose in lignocellulosic hydrolysate by engineered *Yarrowia lipolytica*. *Fermentation.* 8(10). doi: 10.3390/fermentation8100532.

Ding, J., You, S., Ba, W., Zhang, H., Chang, H., Qi, W., et al. (2021). Bifunctional utilization of whey powder as a substrate and inducer for β-farnesene production in an engineered *Escherichia coli*. *Bioresour. Technol.* 341. doi: 10.1016/j.biortech.2021.125739.

Liu, H., Chen, S.-L., Xu, J.-Z., and Zhang, W.-G. (2021). Dual regulation of cytoplasm and peroxisomes for improved α-farnesene production in recombinant *Pichia pastoris*. *ACS Synth Biol.* 10(6), 1563-1573. doi: 10.1021/acssynbio.1c00186.

Liu, Y., Zhang, J., Li, Q., Wang, Z., Cui, Z., Su, T., et al. (2022). Engineering *Yarrowia lipolytica* for the sustainable production of β-farnesene from waste oil feedstock. *Biotechnol. Biofuels Bioprod.* 15(1). doi: 10.1186/s13068-022-02201-2.

Meadows, A.L., Hawkins, K.M., Tsegaye, Y., Antipov, E., Kim, Y., Raetz, L., et al. (2016). Rewriting yeast central carbon metabolism for industrial isoprenoid production. *Nature.* 537(7622), 694-697. doi: 10.1038/nature19769.

Milker, S., and Holtmann, D. (2021). First time β-farnesene production by the versatile bacterium *Cupriavidus necator*. *Microb. Cell Fact.* 20(1). doi: 10.1186/s12934-021-01562-x.

Shi, T., Li, Y., Zhu, L., Tong, Y., Yang, J., Fang, Y., et al. (2021). Engineering the oleaginous yeast *Yarrowia lipolytica* for β‐farnesene overproduction. *J. Biotechnol.* 16(7). doi: 10.1002/biot.202100097.

Yang, S., Vera, J.M., Grass, J., Savvakis, G., Moskvin, O.V., Yang, Y., et al. (2018). Complete genome sequence and the expression pattern of plasmids of the model ethanologen *Zymomonas mobilis* ZM4 and its xylose-utilizing derivatives 8b and 2032. *Biotechnol. Biofuels.* 11, 125. doi: 10.1186/s13068-018-1116-x.

Yao, P., You, S., Qi, W., Su, R., and He, Z. (2020). Investigation of fermentation conditions of biodiesel by-products for high production of β-farnesene by an engineered *Escherichia coli*. *Environ. Sci. Pollut. Res.* 27(18), 22758-22769. doi: 10.1007/s11356-020-08893-z.

You, S., Yin, Q., Zhang, J., Zhang, C., Qi, W., Gao, L., et al. (2017). Utilization of biodiesel by-product as substrate for high-production of beta-farnesene via relatively balanced mevalonate pathway in *Escherichia coli*. *Bioresour Technol.* 243, 228-236. doi: 10.1016/j.biortech.2017.06.058.
